# A 26-Year-Old Female with Systemic Mastocytosis with Associated Myeloid Neoplasm with Eosinophilia and Abnormalities of* PDGFRB*, t(4;5)(q21;q33)

**DOI:** 10.1155/2016/4158567

**Published:** 2016-08-25

**Authors:** Laura E. Brown, Da Zhang, Diane L. Persons, Abdulraheem Yacoub, Shivani Ponnala, Wei Cui

**Affiliations:** ^1^Department of Pathology and Laboratory Medicine, University of Kansas Medical Center, Kansas City, KS, USA; ^2^The Division of Hematologic Malignancies and Cellular Therapeutics, University of Kansas Medical Center, Kansas City, KS, USA

## Abstract

Various translocations involving the* PDGFRB* gene are identified in myeloid neoplasms. However, the* PRKG2/PDGFRB* fusion gene associated with t(4;5)(q21;q33) has previously been reported in only 3 patients. We present the case of a 26-year-old woman with microcytic anemia, basophilia, thrombocytosis, and massive splenomegaly, who was found to have systemic mastocytosis and associated clonal hematological non-mast cell lineage disease (SM-AHNMD), with myeloid neoplasm with* PRKG2/PDGFRB* rearrangement. Initial findings included basophilia (37%, 4.1 k/*μ*L), hypercellular marrow with eosinophilia, and increased and atypical megakaryocytes, suggestive of myeloproliferative neoplasm. Additional studies revealed large clusters of CD25 positive mast cells, fulfilling the criteria for the diagnosis of systemic mastocytosis. Consistent with prior reports of this translocation, our patient has responded well to imatinib. This case, in conjunction with others in the literature, suggests a possible connection between t(4;5)(q21;q33)* PRKG2/PDGFRB* and systemic mastocytosis and highlights their favorable response to imatinib.

## 1. Introduction

Translocations of the platelet-derived growth factor receptor (*PDGFR*) gene are identified in a number of myeloid neoplasms, which are included in the 2008 World Health Organization classification of hematolymphoid tumors as* myeloid and lymphoid neoplasms with eosinophilia and abnormalities of platelet-derived growth factor receptor alpha (PDGFRA), platelet-derived growth factor receptor beta (PDGFRB), and fibroblast growth factor receptor 1 (FGFR1)*. These neoplasms are the result of the fusion genes which result in the production of constitutively active tyrosine kinases, leading to disordered proliferation of hematopoietic tissues [1]. The hallmark feature is the presence of eosinophilia in the bone marrow and peripheral blood. Over 20 fusion partners of* PDGFRB* alone have thus far been identified [2], the most common being fusion of* ETV6* to* PDGFRB*, associated with t(5;12)(q33;p13) [1, 3]. Most patients with* PDGFRB* rearrangements respond well to imatinib therapy [2].

Systemic mastocytosis is a disorder of accumulation of neoplastic mast cells in the bone marrow and other organs, typically associated with organ dysfunction [4]. Frequently systemic mastocytosis is associated with an additional hematological malignancy, termed* systemic mastocytosis associated with clonal hematological non-mast cell lineage disease (SM-AHNMD)*. The associated disorder is most frequently a myeloid neoplasm, including myeloproliferative neoplasm (MPN), myelodysplastic syndrome (MDS), MDS/MPN, and acute myeloid leukemia [4]. Atypical mast cell proliferations or aggregates have been reported in myeloid neoplasms with prominent eosinophilia and abnormalities of* PDGFRA* [5] or* PDGFRB* [6, 7]. Most cases of systemic mastocytosis are associated with a gain-of-function mutation in the* KIT* (CD117) tyrosine kinase, usually a D816V mutation [4]. This mutation confers resistance against imatinib therapy, which is typically only effective in* KIT* wild type patients with systemic mastocytosis [8].

## 2. Case Presentation

**Figure 1:**
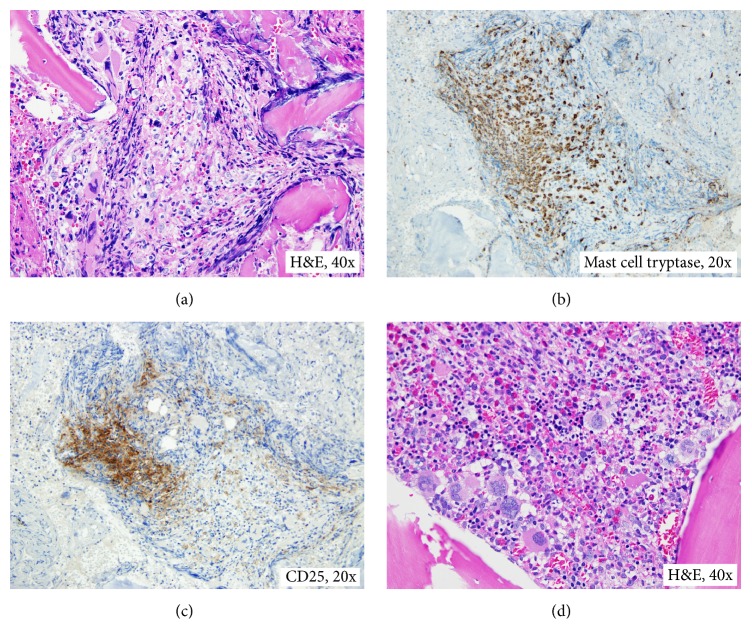
Bone marrow biopsy. (a) Large cluster of mast cells; (b) cluster of mast cells with positive immunohistochemical staining for mast cell tryptase; (c) cluster of mast cells with positive immunohistochemical staining for CD25; and (d) clusters of atypical megakaryocytes.

**Figure 2:**
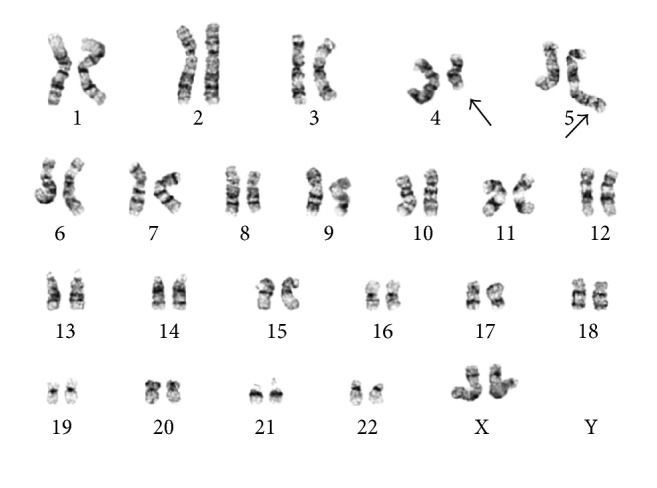
Representative G-banded karyogram showing t(4;5)(q21;q33).

**Figure 3:**
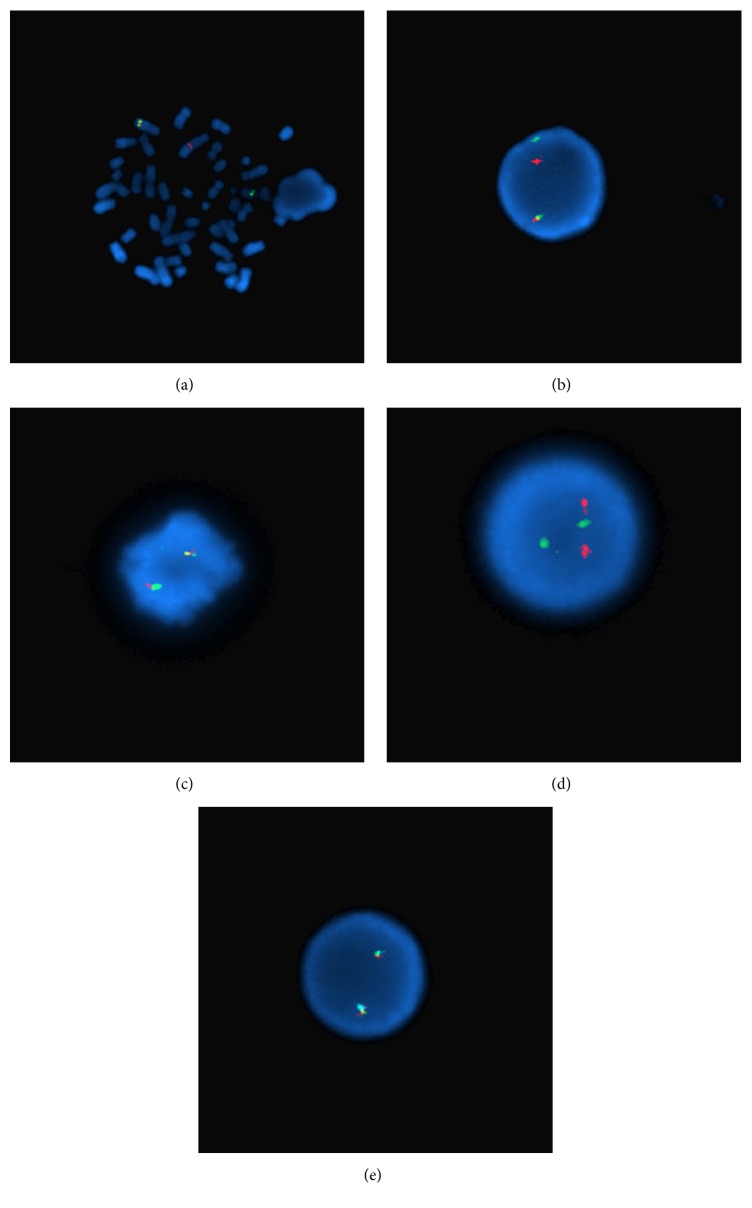
Fluorescence in situ hybridization (FISH) studies. (a) PDGFRB Breakapart Probe (Cytocell, Tarrytown, NY), metaphase illustrating the telomeric green probe translocated to shorter chromosome 4 and the centromeric red probe remaining on elongated chromosome 5; (b) interphase cell illustrating PDGFRB rearrangement, separation of PDGFRB Breakapart Probe; (c) FGFR1 Breakapart/Amplification Probe (Cytocell), normal signal pattern; (d) BCR/ABL1 Translocation, Dual Fusion Probe (Cytocell), normal signal pattern; (e) Vysis (Abbott Molecular, Abbott Park, Illinois) LSI 4q12 Tricolor, Rearrangement Probe, 4q12 (LNX, orange; SCFD2, green; PDGFRA/KIT, aqua), normal signal pattern.

A 26-year-old woman presented with a nearly year-long history of high-grade fever, drenching night sweats, severe fatigue, weight loss of 30% body weight, abdominal pain and distention, and significant skin itching and hives. On examination, she had cachexia and massive splenomegaly. She has no family history of hematologic disorders. Her workup included complete blood count (CBC) that showed hemoglobin of 9.7 g/dL, a platelet count of 764 k/*μ*L, and a leukocyte count of 11.7 k/*μ*L with absolute basophilia (4.1 k/*μ*L) and 2% circulating blasts. Additional workup showed normal iron stores, markedly increased vitamin B12 level (3544 pg/mL), and a serum tryptase level of 175.0 ng/mL. Abdominal imaging confirmed massive splenomegaly (25.9 cm) and hepatomegaly (21.9 cm). On bone marrow examination, the aspiration resulted in a dry tap. Examination of bone marrow trephine biopsy and touch preparations revealed a hypercellular marrow (80–90% cellularity) with increased and atypical hyperlobated megakaryocytes, increased eosinophils (12%), and 2% blasts (Figure 1). A reticulin stain showed mild marrow fibrosis, WHO grade 1/2. Additional studies with immunohistochemical stains for mast cell tryptase and CD117 revealed the presence of large clusters of atypical mast cells (>15 cells), which were positive for CD25 and negative for CD2 (Figure 1). Conventional cytogenetics demonstrated a t(4;5)(q21;q33) in all metaphases and fluorescence in situ hybridization (FISH) confirmed a* PDGFRB* rearrangement demonstrated in Figures 2, 3(a), and 3(b). FISH was negative for* BCR/ABL1*,* PDGFRA*, and* FGFR1* rearrangements (Figures 3(c)–3(e)). KIT Asp816Val mutation analysis performed by qualitative, allele-specific polymerase chain reaction (PCR) assay was negative. Analysis for c.G1849T/p.V617F mutation in exon 14 of JAK2 performed by gene double-dye hydrolysis oligonucleotide probes technology was also negative. Her bone marrow morphologic, immunophenotypic, cytogenetic, and molecular findings were consistent with the diagnoses of* systemic mastocytosis with associated myeloid neoplasm with eosinophilia and abnormalities of PDGFRB, t(4;5)(q21;q33)*.

Based on prior reports of patients with* PRKG2/PDGFRB* mutations and our patient's* KIT* wild type status, she was begun on therapy with 400 mg daily imatinib which was later reduced to 200 mg daily due to side effects and thrombocytopenia. At follow-up approximately 6 months after initiation of imatinib therapy, an excellent clinical response was noted. The patient reported resolution of abdominal pain and distension, as well as of fevers, night sweats, fatigue, and skin manifestations. She achieved a complete hematologic remission. Abdominal imaging showed reduction in the sizes of the spleen to 16.9 cm and the liver to 17 cm. Serum tryptase normalized to 3.9 ng/mL. The patient has not had any additional weight loss and actually gained 5 pounds.

## 3. Discussion 

Cyclic guanosine monophosphate (cGMP) dependent protein kinase type II (*PRKG2*), located at 4q13.1–21.1 [9], is expressed in brain, lung, and intestinal mucosa [10] and is proposed to play a role in intestinal fluid balance and endochondral ossification [10]. The* PRKG2/PDGFR* fusion gene appears to result in the disruption of the juxtamembrane domain of PDGFRB by the PRKG2 coiled coil domain, leading to constitutive activation of tyrosine kinase activity and resulting in dysregulated hematopoiesis [6].

Translocations between* PRKG2* and* PDGFRB* have been described in three other patients to date, both as reciprocal translocations and as part of a more complex rearrangement involving three genes [1, 6, 7]. Common features in patients with this translocation appear to include splenomegaly and mast cell aggregates in the bone marrow [1, 6, 7]. Two patients demonstrated multiple additional features also seen in our case, namely, basophilia, marrow eosinophilia and fibrosis, dysplastic megakaryocytes, and increased serum tryptase [6, 7]. As in our patient, mutational analysis of* KIT* was negative in two of these cases, while in the third the patient was not tested. All three previously reported cases have responded favorably to imatinib therapy, with a symptomatic response occurring in 2 to 12 weeks, hematologic response in 4 to 40 weeks, and cytogenetic remission achieved in 7 to 12 months [1, 6, 7]. In our case the patient has achieved a complete hematologic remission after 6 months.

Our case, in conjunction with those in the literature, suggest an association between t(4;5)(q21;q33)* PRKG2/PDGFRB* and systemic mastocytosis. Previously it has been found that mast cells harbor* PDGFRA* rearrangements in cases of myeloid and lymphoid neoplasms with eosinophilia and abnormalities in* PDFGRA*,* PDGFRB*, or* FGFR1* [11]. Further studies may be of benefit to determine if* PRKG2/PDGFRB* fusion is harbored by mast cells, which would suggest that the cases thus far described may represent a distinct clonal disorder, rather than a systemic mastocytosis with a second non-mast cell hematologic disorder [6]. This case also highlights the fact that patients harboring this translocation are* KIT* wild type and respond favorably to imatinib.
